# Supplementary material for: Sources and Determinants of Discretionary Food Intake in a Cohort of Australian Children Aged 12–14 Months
Source: Int J Environ Res Public Health. 2019 Dec 20;17(1):80. doi: 10.3390/ijerph17010080 (PMC6981432; doi:10.3390/ijerph17010080)
Supplement: Supplementary file 1 [file ijerph-17-00080-s001.pdf]

Supplementary Table 1. Sensitivity analysis: Factors associated with percentage total energy (%TotE) from discretionary foods (mean values and 95% confidence interval) of toddlers with plausible energy intakes (n = 703).

Variables	Unadjusted Mean (%TotE/day)	95% CI	*p*	Adjusted Mean (%TotE/day)	95% CI	*p*
**Total sample**	10.6	10.0 – 11.3				
**Maternal characteristics**						
Maternal age at recruitment (years)			0.078			0.031
<25	12.6	10.4 – 14.8		11.9	9.3 – 14.6	
25–34	10.5	9.8 – 11.3		9.5	8.1 – 10.8	
≥35	9.6	8.3 – 11.0		8.2	6.5 – 9.9	
Level of education			0.001			0.403
High school/vocational	11.7	10.8 – 12.7		10.2	8.4 – 11.9	
Some university and above	9.5	8.7 – 10.4		9.6	8.1 – 11.1	
IRSAD [(a)] score			0.295			
Deciles 1–2 (most disadvantaged)	11.4	9.7 – 13.1				
Deciles 3–4	11.2	9.7 – 12.6				
Deciles 5–6	9.3	7.9 – 10.7				
Deciles 7–8	10.3	8.8 – 11.8				
Deciles 9–10 (most advantaged)	10.7	9.3 – 12.0				
Maternal country of birth			<0.001			<0.001
Australia and New Zealand	11.6	10.8 – 12.3		12.5	11.3 – 13.7	
India	6.4	4.0 – 8.9		7.7	4.9 – 10.5	
China	4.8	2.0 – 7.5		6.9	3.7 – 10.0	
UK	12.3	8.9 – 15.7		13.0	9.4 – 16.6	
Asia Other	5.9	3.5 – 8.3		8.3	5.7 – 10.9	
Other	8.9	6.3 – 11.6		10.8	7.9 – 13.8	
Age of child when mother returned to work			0.223			
≤ 6 months	10.6	9.2 – 12.0				
Between 6 and 12 months	9.7	8.6 – 10.9				
Not returned to work by 12 months	11.0	10.1 – 12.0				
Number of children			0.030			0.022

1	9.8	8.9 – 10.7		8.5	7.1 – 10.0	
2	11.0	9.9 – 12.1		10.2	8.5 – 11.8	
≥3	12.4	10.6 – 14.1		10.9	8.7 – 13.1	
Maternal BMI [(b)] (kg/m²)			0.308			
<25	10.2	9.3 – 11.0				
25–29.99	10.5	9.1 – 12.0				
≥30	11.6	10.0 -13.2				
**Child characteristics**						
Sex						
Male	10.6	9.7 – 11.5				
Female	10.4	9.4 – 11.4				
Age complementary foods introduced (weeks)			0.047			0.663
<17	11.9	10.5 – 13.2		10.2	8.5 – 12.1	
17-25	10.2	9.4 – 10.9		9.6	8.1 – 11.1	
≥26	9.3	7.2 – 11.4		9.7	7.34 – 12.0	

[(a)] IRSAD, Index of Relative Socio-Economic Advantage and Disadvantage, where decile 1 = most disadvantaged and decile 10 = most advantaged. [(b)] BMI Body Mass Index.
